# UK Head and neck cancer surgical capacity during the second wave of the COVID—19 pandemic: Have we learned the lessons? COVIDSurg collaborative

**DOI:** 10.1111/coa.13749

**Published:** 2021-03-29

**Authors:** Richard Shaw, COVIDSurg Collaborative, Andrew G Schache, Michael Wing Sung Ho, Stuart C Winter, James Glasbey, Ian Ganly, Martin Batstone, Juan Rey Biel, Paul C Nankivell, Christian Simon, Omar Omar, Joana FF Simoes, Dmitri Nepogodiev, Aneel Bhangu, Tom Pinkney, Laura McGill, Rita Perry, Terry Hughes, Kwabena Siaw‐Acheampong, Ruth A Benson, Edward Bywater, Daoud Chaudhry, Brett E Dawson, Jonathan P Evans, James C Glasbey, Rohan R Gujjuri, Emily Heritage, Conor S Jones, Sivesh K Kamarajah, Chetan Khatri, Rachel A Khaw, James M Keatley, Andrew Knight, Samuel Lawday, Elizabeth Li, Harvinder S Mann, Ella J Marson, Kenneth A McLean, Siobhan C Mckay, Emily C Mills, Dmitri Nepogodiev, Gianluca Pellino, Maria Picciochi, Elliott H Taylor, Abhinav Tiwari, Joana FF Simoes, Isobel M Trout, Mary L Venn, Richard JW Wilkin, Aneel Bhangu, James C Glasbey, Richard Shaw, Andrew G Schache, Stuart C Winter, Michael WS Ho, Paul Nankivell, Juan Rey Biel, Martin Batstone, Ian Ganly, Christian Simon, Joana FF Simoes, Tom EF Abbott, Michel Adamina, Adesoji O Ademuyiwa, Arnav Agarwal, Ehab Alameer, Derek Alderson, Felix Alakaloko, Markus Albertsmeiers, Osaid Alser, Muhammad Alshaar, Sattar Alshryda, Alexis P Arnaud, Knut Magne Augestad, Faris Ayasra, José Azevedo, Brittany K Bankhead‐Kendall, Emma Barlow, Ruth A Benson, Ruth Blanco‐Colino, Amanpreet Brar, Ana Minaya‐Bravo, Kerry A Breen, Chris Bretherton, Igor Lima Buarque, Joshua Burke, Edward J Caruana, Mohammad Chaar, Sohini Chakrabortee, Peter Christensen, Daniel Cox, Moises Cukier, Miguel F Cunha, Giana H Davidson, Anant Desai, Salomone Di Saverio, Thomas M Drake, John G Edwards, Muhammed Elhadi, Sameh Emile, Shebani Farik, Marco Fiore, J Edward Fitzgerald, Samuel Ford, Tatiana Garmanova, Gaetano Gallo, Dhruv Ghosh, Gustavo Mendonça Ataíde Gomes, Gustavo Grecinos, Ewen A Griffiths, Madalegna Gründl, Constantine Halkias, Ewen M Harrison, Intisar Hisham, Peter J Hutchinson, Shelley Hwang, Arda Isik, Michael D Jenkinson, Pascal Jonker, Haytham MA Kaafarani, Angelos Kolias, Schelto Kruijff, Ismail Lawani, Hans Lederhuber, Sezai Leventoglu, Andrey Litvin, Andrew Loehrer, Markus W Löffler, Maria Aguilera Lorena, Maria Marta Madolo, Piotr Major, Janet Martin, Hassan N Mashbari, Dennis Mazingi, Symeon Metallidis, Ana Minaya‐Bravo, Helen M Mohan, Rachel Moore, David Moszkowicz, Susan Moug, Joshua S Ng‐Kamstra, Mayaba Maimbo, Milagros Niquen, Faustin Ntirenganya, Maricarmen Olivos, Kacimi Oussama, Oumaima Outani, Marie Dione Parreno‐Sacdalanm, Francesco Pata, Carlos Jose Perez Rivera, Thomas D Pinkney, Willemijn van der Plas, Peter Pockney, Ahmad Qureshi, Dejan Radenkovic, Antonio Ramos‐De la Medina, Keith Roberts, April C Roslani, Martin Rutegård, Irène Santos, Sohei Satoi, Raza Sayyed, Andrew Schache, Andreas A Schnitzbauer, Justina O. Seyi‐Olajide, Neil Sharma, Richard Shaw, Sebastian Shu, Kjetil Soreide, Antonino Spinelli, Grant D Stewart, Malin Sund, Sudha Sundar, Stephen Tabiri, Philip Townend, Georgios Tsoulfas, Gabrielle H van Ramshorst, Raghavan Vidya, Dale Vimalachandran, Oliver J Warren, Duane Wedderburn, Naomi Wright, EuroSurg, European Society of Coloproctology (ESCP), Global Initiative for Children’s Surgery (GICS), GlobalSurg, GlobalPaedSurg, ItSURG, PTSurg, SpainSurg, Italian Society of Colorectal Surgery (SICCR), Association of Surgeons in Training (ASiT), Irish Surgical Research Collaborative (ISRC), Transatlantic Australasian Retroperitoneal Sarcoma Working Group (TARPSWG), Italian Society of Surgical Oncology (SICO), LA Boccalatte, M Batstone, R Hodge, J Abeloos, T De Backer, J De Ceulaer, C Dick, A Diez‐Fraile, P Lamoral, C Spaas, DLAL Schrijvers, EBM Willemse, C Faris, S Maariën, G Van Haesendonck, C Van Laer, P Deron, EA Abdallah, GB Carvalho, L Kowalski, J Vartanian, AP Gatti, CN Nardi, RNL Oliva, MC Salem, D Cheng, D MacNeil, J Martin, R Mayer, G Groot, L Acosta, M Mejia, CJ Perez, M Lorencin, I Luksic, M Mamic, FM Ashoush, NA Osman, M Safwat Shahine, A Eldaly, MMA Elfiky, A Amin, R Elmorsi, B Refky, MM Essa, Mengistu G Mengesha, S Dakpé, S Boucher, Q Ballouhey, J Laloze, J Usseglio, C Hoffmann, V Gregoire, B Lallemant, M Blaurock, D Reim, A Boehm, O Guntinas‐Lichius, F Hölzle, A Modabber, P Winnand, J Kleeff, K Lorenz, U Ronellenfitsch, R Schneider, CS Betz, A Böttcher, C Busch, N Möckelmann, JM Inhestern, J Greve, TK Hoffmann, S Laban, JM Vahl, A Agyeman‐Prempeh, D Aning, I Barnor, R Darko‐Asante, M Dzogbefia, V Gaveh, D Gyimah, MD Issahalq, A Konney, M Poku, T Adjeso, ET Akornor, WO Amankwaa, DA Antwi, P Apppiah‐Thompson, M Damah, EO Kumi, L Manan, JP Murphy, L Osei, J Setuagbe, N Arkadopoulos, N Danias, P Economopoulou, M Frountzas, P Kokoropoulos, A Larentzakis, NV Michalopoulos, K Nastos, S Parasyris, E Pikoulis, J Selmani, TA Sidiropoulos, P Vassiliu, CE Kalfountzos, I Chatziioannou, C Corais, E Gkrinia, A Ntziovara, A Saratziotis, K Antoniadis, O Orestis, D Tatsis, E Baili, A Charalabopoulos, T Liakakos, D Schizas, E Spartalis, A Syllaios, C Zografos, M Aguilera‐Arévalo, S Misra, P Pareek, J Vishnoi, P Chappity, M Kar, DK Muduly, M Sultania, S Agarwal, PK Garg, DD Maharaj, KS Majumdar, N Mishra, D Poonia, RK Seenivasagam, MP Singh, AR Tiwari, P Penumadu, S Rajan, S Kumar, R Raychowdhury, R Ghodke, R Raychowdhury, C Barry, D Callanan, A Dias, L Haung, A Ionescu, P Sheahan, P Lennon, A Mizrachi, A Deganello, R Pellini, B Pichi, F Lemma, MV Marino, M Bergonzani, A Varazzani, F Bussu, T Perra, A Piras, A Porcu, D Rizzo, G Campisi, A Cordova, M Franza, G Rinaldi, F Toia, A Gianni, L Giannini, L Gordini, E Baldini, L Conti, A De Virgilio, F Ferreli, F Gaino, G Mercante, G Spriano, M Ansarin, F Chu, R De Berardinis, G Ietrobon, M Tagliabue, F Ionna, A Baietti, P Maremonti, F Neri, G Prucher, S Ricci, A Casaril, M Nama, A Cotoia, V Lizzi, F Vovola, P Bruzzaniti, P Familiari, P Lapolla, G Marruzzo, A Mingoli, D Ribuffo, R Cipriani, F Contedini, M Lauretta, C Marchetti, M Melotti, M Pignatti, V Pinto, A Pizzigallo, F Ricotta, A Tarsitano, L Catarzi, G Consorti, EA Abdulwahed, EA Alshareea, MI Biala, RJ Ghmagh, AF Ibrahim, YT Liew, MR Alvarez, R Arrangoiz, F Cordera, A Gómez‐Pedraza, CE Soulé‐Martínez, OS Becerril, GFC Becerra, Y Arkha, H Bechri, A El Ouahabi, M Oudrhiri, A Benkabbou, M Majbar, R Mohsine, A Souadka, N Lageju, WH Schreuder, J Hardillo, R de Bree, D Schweitzer, AA Adeyeye, EE Enoch, TSTT Sholadoye, F Wuraola, O Oyelakin, MI Khokhar, B Ayub, M Walędziak, M Szewczyk, C Faria, P Cardoso, J Castro Silva, E AlKharashi, D Jelovac, M Petrovic, S Sumrak, RR Asceric, JM Bojicic, BM Kovacevic, ID Krdzic, MA Milentijevic, VZ Milutinovic, ZB Stefanovic, JM Villacampa, V Jiménez Carneros, A Salazar Carrasco, A Carabias Hernandez, L Alonso Lamberti, R León Ledesma, FJ Jiménez Miramón, JM Jover Navalón, J Garcia Quijada, J Ramos Rodriguez, A Valle Rubio, I Vilaseca, J Escartin, M Estaire‐Gomez, D Padilla Valverde, M Tousidonis, F Lopez, U Deandrés‐Olabarria, M Durán‐Ballesteros, F Fernández‐Pablos, F Ibáñez‐Aguirre, A Sanz‐Larrainzar, B Ugarte‐Sierra, M Di Martino, J Prada, UM Jariod‐Ferrer, A Landaluce Olavarria, J Rey‐Biel, P Díaz de Cerio, A Sánchez Barrueco, EK Lindqvist, M Sund, R Piantanida, R Giger, S Hool, SA Müller, SJ Stoeckli, C Simon, T Toutounji, A Al assaf, AM Hammed, SM Hammed, M Mahfoud, A Arikan, Ö Yalkin, N İflazoğlu, A Isik, S Leventoglu, L Aydemir, B Basaran, C Sen, M Comert–Ulusan, B Basaran, KT Saracoglu, A Saracoglu, B Mantoglu, G Kucuk, N Aygun, E Baran, M Tanal, A Eray Tufan, M Uludag, S Gürkan Yetkin, B Yigit, B Calik, S Demirli Atici, T Kaya, FK Sikakulya, KMAH Abdel‐Galil, T Lowe, AJ Durrani, A Habeeb, E Irune, L Luke, L Masterson, SH Murphy, N Segaren, C Walker, S Waseem, TM Jones, C Loh, S Pringle, AG Schache, RJ Shaw, J Stenhouse, M Armstrong, S Sood, D Sutton, S Thomas, P Clarke, SC Winter, S Hislop, PR Counter, N Ghazali, C Lloyd, V Prabhu, D Godden, S Whitley, C Butler, R Nash, K El‐Boghdadly, A Fry, R Niziol, M De, CK Gill, S Crank, AD Mace, M Ho, M Mair, P Kothari, J Homer, S Sainuddin, RJ Egan, M Kittur, C Burgess, J O'Hara, J Manickavasagam, C McDonald, S Burrows, KR Java, C Katre, A Ahmed, H Siddique, E King, P Ramchandani, PR Naredla, P Brennan, T Ringrose, F Schmidt, JKC Mak, P Nankivell, S Parmar, N Sharma, C Douglas, J McCaul, J McCaul, J Dhanda, N Ghazali, P Kyzas, L Vassiliou, A Kumar, A Husband, J Hulbert, D Ingrams, R Parkin, I Varley, D Gahir, A George, D Zakai, M Bater, C Surwald, B Devlin, CG Leonard, N Pigadas, D Snee, RP Singh, NC Hyde, M Paley, H Cocks, A Wilson, D Choi, CJ Kerawala, F Riva, A Dickason, CJ Semple, C Schilling, PR Naredla, G Walton, O Rees‐Stoner, N Scott, IJ Nixon, D Tighe, S Mattine, MMH Chu, V Pothula, W Lee, L Brown, I Ganly, N Alpert, CN Illezeau, B Miles, J Rapp, E Taioli, MT Azam, AJ Choudhry, W Marx, J Stein, Y Ying, ND Gross, M Almasri, R Joshi, G Kulkarni, H Marwan, M Mehdi, B Sumer

**Affiliations:** ^1^ Liverpool Head & Neck Centre The University of Liverpool Cancer Research Centre Liverpool UK

**Keywords:** capacity, COVID‐19, critical care, delay, head and neck cancer, pandemic, SARS‐CoV‐2, surgery

## Abstract

**Objectives:**

The aim of this study was to evaluate the differences in surgical capacity for head and neck cancer in the UK between the first wave (March‐June 2020) and the current wave (Jan‐Feb 2021) of the COVID‐19 pandemic.

**Design:**

REDcap online‐based survey of hospital capacity.

**Setting:**

UK secondary and tertiary hospitals providing head and neck cancer surgery.

**Participants:**

One representative per hospital was asked to report the capacity for head and neck cancer surgery in that institution.

**Main outcome measures:**

The principal measures of interests were new patient referrals, capacity in outpatients, theatres and critical care; therapeutic compromises constituting delay to surgery, de‐escalated surgery and therapeutic migration to non‐surgical primary modality.

**Results:**

Data were returned from approximately 95% of UK hospitals with a head and neck cancer surgery specialist service. 50% of UK head and neck cancer patients requiring surgery have significantly compromised treatments during the second wave: 28% delayed, 10% have received radiotherapy‐based treatment instead of surgery, and 12% have received de‐escalated surgery. Surgical capacity has been more severely constrained in the second wave (58% of pre‐pandemic level) compared with the first wave (62%) despite the time to prepare.

**Conclusions:**

Some hospitals are overwhelmed by COVID‐19 and unable to offer essential cancer surgery, but all have neighbouring hospitals in their region retaining good (or even normal) capacity. It is noteworthy that very few patients have been appropriately redirected away from the hospitals most constrained by their burden of COVID‐19. The paucity of an effective central or regional strategic response to this evident mismatch between demand and surgical capacity is to the detriment of our head and neck cancer patients.

**Keypoints**

‐ 1‐week survey, 1 to 8 February 2021, with 62 UK HN cancer surgery hospitals responding, indicating around 95% response rate
‐ 50% of UK HN cancer patients requiring surgery currently having compromised treatment: 28% delayed, 12% de‐escalated, 10% received radiotherapy instead.
‐ In the worst third of hospitals, 82% of HN cancer patients needing surgery had compromised treatment.
‐ This restriction in capacity is no better than the first wave response, despite advanced warnings for winter 2020/21, and 6 months lead time to prepare the NHS strategic response.
‐ Hospitals with HN surgical capacity particularly badly affected by COVID‐19 all have neighbouring units with very good capacity—but patients have not generally been redirected
‐ New HN cancer referrals have picked up from 65% to 80% of pre‐COVID level, with some units reporting >100%
‐ Urgent action is needed at central strategic level to manage the “bow wave” of cancers still awaited

## 1 OBJECTIVES

For head and neck (HN) cancers treated by surgery in the first wave of the COVID‐19 pandemic between March and June 2020, it was evident that surgical and critical care capacity was greatly reduced. As a consequence of these constraints, as well as concerns over cross‐infection with SARS‐CoV‐2, HN surgery was minimised, de‐escalated or avoided in many centres.^1^ Additionally, many patients underwent therapeutic migration away from primary surgery.^2^ The consequences for oncology and functional outcomes are unknown but form the focus for an international follow‐up to COVIDSurg‐HN funded by BAHNO (The British Association of Head and Neck Oncologists). DATA‐CAN estimates^3^ that UK HN cancer referrals fell by up to 60% during the first COVID‐19 spike, building a bow wave of undiagnosed upstaging cases. Amongst other legacies of the COVID‐19 pandemic, increased mortality and compromised functional outcomes for HN cancer patients seem certain. The COVIDSurg collaborative established that pulmonary complications and mortality were unacceptably high in postoperative patients who contracted SARS‐CoV‐2 infection,^4^ but data on the safety of HN surgery, even when complex and prolonged, proved comparatively reassuring. Risk in elective cancer surgery was reduced by around 50% where COVID‐19‐free care pathways were employed,^5^ and COVID‐19 occurred after HN surgery in only 3% of cases,^2^ with an all‐cause 30‐day mortality of 1.2%.

A severe second wave of COVID‐19 during winter 2020/21 was indeed widely predicted, even during the first wave.^6^ A plea was made for governments to act through preparation of labour, resources and facilities to reduce the burden, not only on COVID‐19 mortality, but also for other life‐limiting conditions including cancer.^6^ Understanding that the backlog in HN cancer is incompletely resolved, second‐wave surgical capacity appears particularly critical. The reassuring data on safety in the first wave reinforce that with appropriate testing, PPE and cross‐infection measures, HN cancer surgery should continue without fear of excess risk, even through a period of very high community COVID‐19 incidence.

The aim of this study was to evaluate the differences in surgical capacity for head and neck cancer in the UK between the first (March‐June 2020) and the current (Jan‐Feb 2021) COVID‐19 pandemic waves. Further, we report on efforts in strategic planning and mutual aid between hospitals.

## 2 DESIGN

REDCap (Research Electronic Data Capture) was employed to record all data through its web application. A 13‐item survey questionnaire (Appendix S2) was constructed according to established survey design and conduct guidance.^7^, ^8^ Both pre‐testing and pilot testing evaluation of the questionnaire was undertaken by members of the COVIDSurg‐HN writing group prior to survey distribution.

## 3 SETTING AND PARTICIPANTS

A confidential survey was distributed electronically to existing COVIDSurg‐HN collaborative members, a wide group which had collected in excess of 5000 head and neck cancer treatments carried out during March‐June 2020.^2^ All UK HN specialty associations were contacted as well as using direct social media. Respondents were requested to provide a single response on behalf of their hospital. Duplicates were reviewed and averaged for subsequent analysis. The UK responses were compared with a list of all units providing HN cancer surgery (DAHNO).^9^ Each participating hospital's data referred to the management of adult patients undergoing HN cancer surgery with curative intent.

## 4 MAIN OUTCOME MEASURES

The survey period was from 1 to 7 February 2021. Respondents were requested to complete the survey with estimates of HN cancer service capacity at three distinct timepoints:

- prior to the COVID‐19 pandemic.
- during the first peak of COVID‐19 infection (March‐June 2020).
- during the current peak of COVID‐19 (January‐February 2021).

The entry fields represented either percentage estimates, defined response criteria or direct yes/no responses. In all instances, these responses were classified as required fields to complete the survey. Data collected included the geographical location of the hospital, access to operating lists, face‐to‐face versus virtual outpatient clinic attendance and degree of access to critical care beds following HN cancer surgery. Data were collected regarding operating capacity at alternative hospital sites, and if so whether surgery performed by a differing surgical team. We ascertained the extent of delay to HN cancer surgery beyond 62‐day rule, surgical de‐escalation and therapeutic migration away from primary surgery. No specific reporting guideline was followed.

## 5 RESULTS

Data were obtained from 73 individual returns but were incomplete for one hospital. Ten hospitals had (understandably) entered duplicate data given the multiple channels of communication employed. In most cases, the duplicate data returned were very closely matched and for others represented a difference in caseload between the ENT and OMFS surgeons entering their own data, so a crude mean of the two data entries was employed in these cases. By comparison with the DAHNO list^9^ of HN surgery units, a further three UK centres did not provide data, providing a best estimate for survey response rate of 95.5%. Following data cleaning, complete data on surgical capacity for head and neck cancer for the 62 hospitals are presented.

### 5.1 Data summary

Estimates of HN cancer referrals are currently (Jan‐Feb 2021) on average, at 85% of pre‐pandemic levels (range 35%‐120%) compared with mean 61% (range 20%‐100%) during the first wave in March‐June 2020. Notably, 5 of the 62 (12%) units report an even higher current caseload than prior to the COVID‐19 pandemic, a feature not apparent in the first wave in any centre. Theatre capacity was slightly lower in the second wave, estimated as a mean 58% (Jan‐Feb 2021) versus 62% (March‐June 2020) of pre‐pandemic levels. Outpatient appointments carried out face‐to‐face increased to a mean 65% during the second wave, from a mean 46% in the first wave and 98% prior to the pandemic. 26% of HN surgery teams have had no access to critical care beds during the second wave, and 42% have substantially reduced access. Comparable data for the first wave are 15% and 44%, although this is in the context that the majority of hospitals (62%, 38/62) reporting occasional difficulty in obtaining critical care beds for HN cancer patients prior to the pandemic. In the second wave, 30% of HN teams have had at least some access to other hospital sites to alleviate pressure from their “usual” hospital site, although in only 11% has this required transfer of patient care for surgery to be performed by another team. This has remained consistent throughout differing periods of the pandemic, with comparable data for the first wave being 40% and 10%, respectively. Surgery has been delayed in a mean 28% of HN cancer patients during the second wave, very similar to 30% in the first wave. Pre‐pandemic delay was only 6% on average. A switch in therapeutic modality away from primary surgery is reported in 10% of second‐wave cases, and surgical de‐escalation in 12% second wave, compared with 20% and 17%, respectively, during the first wave.

### 5.2 Categorisation by current surgical capacity

Hospitals were categorised by current operating theatre capacity for HN cancer: “*normal*” > = 100% pre‐pandemic theatre capacity (15 units, 24%), “*reduced*” > = 50% and <100% (26 units, 42%); “*greatly reduced*” <50% (21 units, 34%). These data are summarised in Table 1. For those units reporting greatly reduced surgical capacity, most also had no access to CCU, and delay to surgery beyond 62 days was estimated in a mean of 46% of cases, with therapeutic compromise in 82% of cases. In contrast, hospitals with normal level of capacity understandably had a much lower index of surgical compromise (delay 4%, compromise 10%).

**TABLE 1:** Therapeutic impact to HN cancer surgery categorised by degree of reductions in capacity during Jan/Feb 2021

Jan/Feb 2021 Theatre capacity			Jan/Feb 2021 CCU access for HN surgery				Therapeutic compromises				Alternative Surgical site used?	
Category	Value	Number of Units	Unrestricted	Occasionally restricted	Reduced Capacity	No access to CCU	Surgery delayed >62 d	De‐escalated surgery	Therapeutic migration to Radiotherapy/CRT	TOTAL Compromised cases	Other site, same team	Different operating team
Total	All	**62 (100%)**	**9 (15%)**	**11 (18%)**	**26 (42%)**	**16 (26%)**	**28%**	**12%**	**10%**	**50%**	**18 (29%)**	**7 (11%)**
Normal	> = 100%	15 (24%)	6	2	5	2	4%	6%	0%	**10%**	2 (13%)	0 (0%)
Reduced	> = 50% <100%	26 (42%)	2	9	12	3	28%	13%	8%	**49%**	6 (23%)	2 (8%)
Greatly Reduced	<50%	21 (34%)	1	0	9	11	46%	16%	20%	**82%**	10 (48%)	5 (24%)

The bold was merely to highlight/ signify totals.

### 5.3 Categorisation by geographical region

**FIGURE 1:**
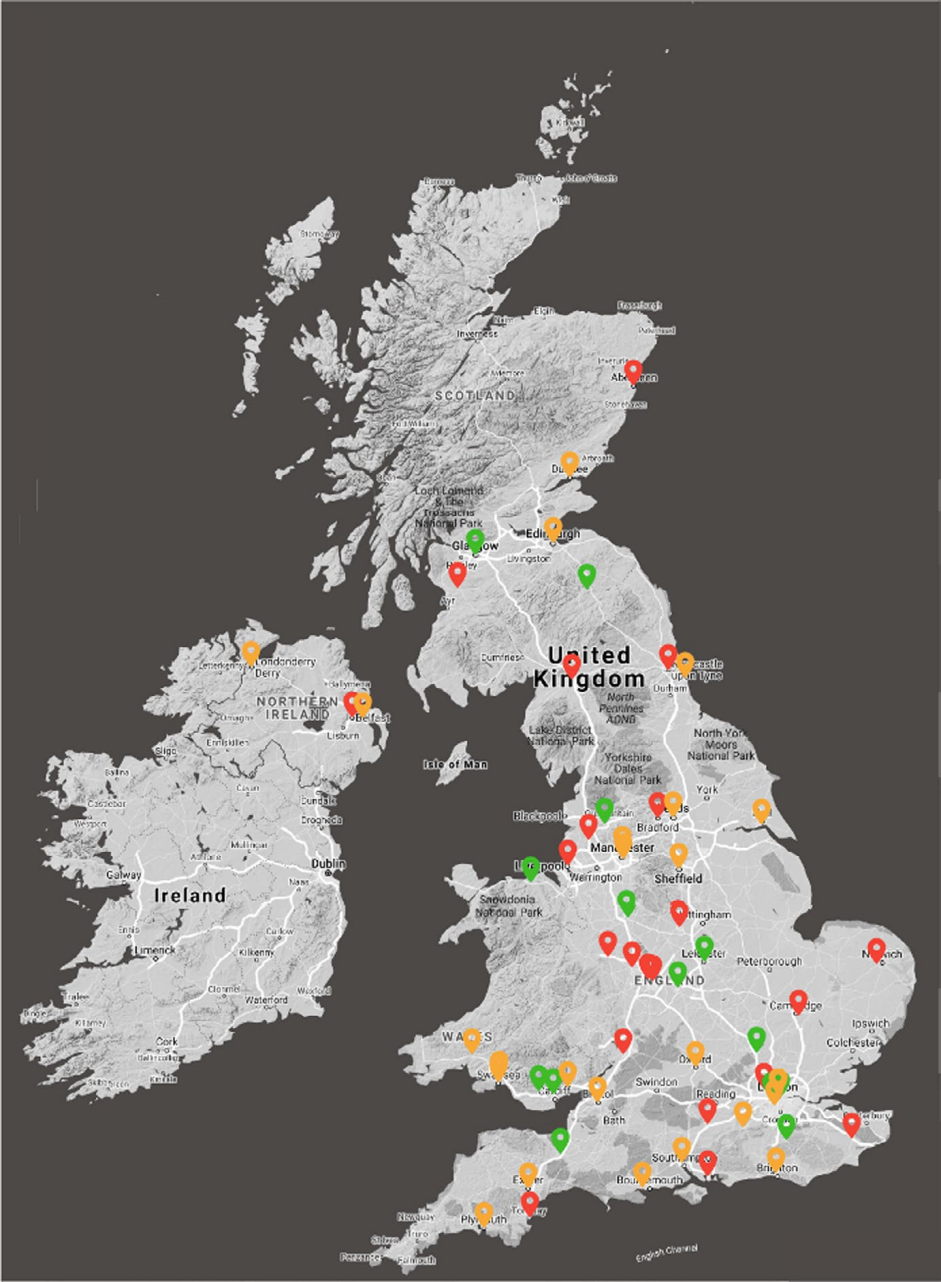
Proximity of UK HN surgical units with normal >=100% (green), reduced >=50% and < 100% (amber) and greatly reduced < 50% (red) surgical capacity during 2nd wave COVID‐19 pandemic Jan / Feb 2021

UK hospitals were categorised geographically into 10 regions, and their data comparing the impact of surgical capacity on therapeutic compromise are presented in Table 2. The regions reported broadly similar surgical capacity, ranging between 40% and 75% of pre‐pandemic levels. However, the impact on therapeutic compromise differed greatly by region: delay to surgery between 2% and 62% of patients; surgical de‐escalation between 0% and 23%; and therapeutic migration between 0% and 40% of patients. Regions hosting hospitals with surgical capacity that were particularly affected by COVID‐19 had neighbouring units with very good capacity maintained (Figure 1). This did not appear to result in movement of cases between sites nor minimise therapeutic compromises. The distribution of surgical capacity therefore did not align well with demand. The 6 units with less than 5% of their pre‐pandemic levels of access to operating theatres were spread amongst 5 geographic regions. None of these had any current access to CCU, half did not have access to operate on alternative sites and two‐thirds had not referred patients elsewhere.

**TABLE 2:** Therapeutic impact to HN cancer surgery categorised by region during Jan/Feb 2021

		Theatre Capacity For HN cancer surgery % of pre‐pandemic level		Therapeutic Compromises					
				Delay >62 D		De‐escalation of surgery		Therapeutic migration from surgery to RT/CRT	
Region	Number of Centres	Mean capacity	Range	Mean	Range	Mean	Range	Mean	Range
**All**	**62**	**58%**	**0%‐100%**	**28%**		**12%**		**10%**	
SE & London	13	75%	30%‐125%	20%	0%‐80%	7%	0%‐50%	20%	0%‐80%
East	3	50%	20%‐100%	60%	0%‐100%	8%	0%‐25%	18%	10%‐25%
South	5	53%	0%‐95%	23%	0%‐150%	10%	0%‐30%	5%	0%‐15%
South West	6	52%	0%‐100%	19%	0%‐50%	0%	n/a	0%	n/a
Midlands	7	40%	0%‐100%	62%	0%‐100%	22%	0%‐70%	40%	0%‐100%
North West	5	49%	0%‐100%	14%	0%‐25%	1%	0%‐5%	5%	0%‐25%
Yorkshire & North	7	47%	25%‐100%	31%	0%‐80%	17%	0%‐40%	3%	0%‐20%
Scotland	6	60%	0%‐100%	2%	0%‐10%	8%	0%‐50%	0%	0%‐0%
Wales	7	75%	50%‐100%	26%	0%‐50%	20%	0%‐100%	17%	0%‐50%
N. Ireland	3	49%	10%‐85%	53%	10%‐100%	23%	0%‐50%	7%	0%‐20%

The bold was merely to highlight/ signify totals.

## 6 DISCUSSION

These data show that surgical capacity to treat HN cancer has again been severely impacted during the second wave of COVID‐19 during January and February 2021. The extent of its impact is broadly comparable to the first wave, with one‐third of hospitals with greatly reduced theatre capacity, a quarter with zero access to postoperative critical care. Overall, half of HN cancer patients requiring surgery have compromised treatments. The national and regional average data, however, obscure important and even more troubling detail. A minority of hospitals have been completely overwhelmed by patients suffering with COVID‐19 such that they are unable to offer any meaningful HN surgery despite recovering referral pathways and resultantly increasing case demand. Unfortunately, the NHS has not prepared for redirection of patients away from such hospitals where, as a result, the compromises to effective and timely therapy are most significant.

This study has been conceived, data have been collected, cleaned and analysed, written, peer‐reviewed and pre‐published within a 2‐week window. The penetration to UK HN surgery centres has been greater than 95%, and colleagues have returned data irrespective of regional or specialty allegiances. Once again, the medical profession has stepped up to the challenges of COVID‐19 and rapidly established new ways to work and collaborate, as amply demonstrated by the COVIDSurg^2^, ^4^, ^5^ collaborative effort. Admittedly, all data collected reflect the respondents’ best estimates and some of the analyses would be best regarded semi‐quantitatively, or as qualitative impressions. There has been no attempt to weigh the data by size of unit or HN cancer caseload. There is a risk of false precision in “over‐analysis” of the reported numerical data, and consequently, detailed statistical analyses were unwarranted.

It appears that the UK second COVID‐19 wave is somewhat more severe than the first, as evidenced by daily announced death rates reaching 1820 early in 2021,^10^ and COVID‐19 patients NHS bed occupancy at around 150% of first wave. Our data show that access to critical care for surgical oncology is correspondingly worse in the second wave (no access: 26%, reduced access: 42%) than the first wave (no access: 18%, reduced access: 47%). This is despite efforts to expand intensive care facilities, often at the expense of elective capacity. In this context, the ability to sustain only marginally reduced HN cancer theatre capacity (58% vs 62%) is evidence that within individual hospital trusts mitigations to maintain surgical oncology have been partly effective. However, new HN cancer referrals have picked up from 65% to 80%, with some units reporting higher demand than normal pre‐pandemic levels. Interestingly, virtual consultations have dropped 65% to 46%. It is speculated that surgeon and patient dissatisfaction may be responsible for this fall off, or possibly safety concerns regarding missing cancers diagnoses without clinical examination in face‐to‐face clinics.

Despite 6‐month preparation time between the waves, half of cancer treatments have been significantly compromised during the second wave (first wave: 30% delay, 17% RT, 20% de‐escalated,total 67% versus second wave 28%, 10%, 12%, total 50%). Transfer of care to other teams remains uncommon. The 10% figure reflects data per hospital and presumably translates into a rather lower percentage of individual patients actually being transferred. Surgeons moving their service to operate at another site is a common mitigation in first and second wave and presumably reflects use of the private sector or mutual aid from “cold” sites. Of note, surgeons were only half as likely to accept de‐escalation 20% vs 12%, similarly less likely to accept therapeutic migration 17% vs 10%, during the second wave. As intuition would predict, units with most severely affected surgical capacity also experience the greatest extent of therapeutic compromise (82% vs 49% vs 10% in those hospitals with greatest reduction, reduced and normal capacity, respectively). For a third of hospitals treating HN cancer, their greatly reduced capacity reflects a major detrimental effect on cancer treatment, with likely significant consequent implications for mortality. Critically, hospitals with most severely constrained surgical capacity all do appear to have neighbouring units in the same region maintaining good or near‐normal capacity, but this has unfortunately not translated into widespread transfer of patients.

In the UK, the lead time to prepare for this second wave was around 5‐6 months, and with the National Health Service under central strategic control, it was presumably plausible, and certainly highly desirable, to optimise the available resources. This would have minimised consequent rationing of, and delay to, primary surgery for HN cancers. Since the start of the pandemic, international and UK experience has repeatedly shown that the major impacts of COVID‐19 fall disproportionately upon certain regions and at certain times. Anecdotally, one major UK HN unit has been hosted by a hospital burdened by more than 1000 inpatient beds taken by COVID with 200 of them ventilated. Where this impact coincides with a large volume HN surgery centre, perhaps redirection of patient pathways could be very effective and should be deployed. The mismatch between supply and demand appears to be severe in a significant minority of hospitals but has not yet been properly addressed at a central strategic level, perhaps reflecting lack of capacity and authority to implement such decisive actions. Which bodies have both the required data *and* the authority to implement redistribution of cancer cases for best interests of our patients: cancer alliances, NHS trusts, national associations? It is not clear to what extent is HN surgery typical of all surgical oncology in this respect. HN surgery has a particularly onerous requirement for airway skills, specialist nursing and professions allied to medicine and bulky, expensive and fragile specialist equipment such as microscopes, plating kits, lasers and robots. This makes moving away from COVID‐19 affected sites logistically complex. It is also possible that some surgeons make such a personal interconnection with their patients early in their care pathway, that there may be a cultural resistance to accept another team could achieve similar results, and a consequent reluctance to refer on. Conversely, patients may also choose to remain with a team they have met or have difficulty travelling to a hospital further afield.

These issues reveal considerable fragility within the NHS, where life‐critical but elective services can apparently be so vulnerable to acute emergency pressures. This lack of resilience in elective care is often seen to a lesser extent as “winter pressures,” but not nearly as severely as in the current second COVID‐19 wave. The lack of national strategic response is of great concern, but it is perhaps not yet too late to act. The “bow wave” of delayed HN cancer cases remains ahead of us and many more months of COVID‐19 restrictions remain.

## CONFLICT OF INTEREST

The authors have no conflicts of interest to declare.

## ETHICAL APPROVAL

No ethical approval was obtained as confidentiality of responders / hospitals, and anonymity of data presentation was maintained. Other than encouragement to contribute to better understanding of the impact of COVID‐19 on Head & Neck services, there were no incentives to respond. The authors have no conflicts of interest to declare.

## Supporting information

Supplementary Material

Supplementary Material

## Data Availability

Anonymised source data are available by contacting BiCOPS through the corresponding author.

## 1 Authorship COVIDSurg Collaborative*

### 1.1 Collaborators (PubMed‐citable)

#### 1.1.1 Writing group

Richard Shaw, Andrew G Schache, Michael Wing Sung Ho, Stuart C Winter, James Glasbey, Ian Ganly, Martin Batstone, Juan Rey Biel, Paul C Nankivell, Christian Simon, Omar Omar, Joana FF Simoes, Dmitri Nepogodiev, Aneel Bhangu, Tom Pinkney, Laura McGill, Rita Perry, Terry Hughes

#### 1.1.2 CovidSurg Operations Committee

Kwabena Siaw‐Acheampong, Ruth A Benson, Edward Bywater, Daoud Chaudhry, Brett E Dawson, Jonathan P Evans, James C Glasbey, Rohan R Gujjuri, Emily Heritage, Conor S Jones, Sivesh K Kamarajah, Chetan Khatri, Rachel A Khaw, James M Keatley, Andrew Knight, Samuel Lawday, Elizabeth Li, Harvinder S Mann, Ella J Marson, Kenneth A McLean, Siobhan C Mckay, Emily C Mills, Dmitri Nepogodiev, Gianluca Pellino, Maria Picciochi, Elliott H Taylor, Abhinav Tiwari, Joana FF Simoes, Isobel M Trout, Mary L Venn, Richard JW Wilkin, Aneel Bhangu.

#### 1.1.3 International Cancer Leads (*denotes specialty Principal Investigator)

James C Glasbey (*Chair*); Head and neck: Richard Shaw*, Andrew G Schache, Stuart C Winter, Michael W S Ho, Paul Nankivell, Juan Rey Biel, Martin Batstone, Ian Ganly, Christian Simon.

#### 1.1.4 Dissemination Committee

Joana FF Simoes (*Chair*); Tom EF Abbott, Michel Adamina, Adesoji O Ademuyiwa, Arnav Agarwal, Ehab Alameer, Derek Alderson, Felix Alakaloko, Markus Albertsmeiers, Osaid Alser, Muhammad Alshaar, Sattar Alshryda, Alexis P Arnaud, Knut Magne Augestad, Faris Ayasra, José Azevedo, Brittany K Bankhead‐Kendall, Emma Barlow, Ruth A Benson, Ruth Blanco‐Colino, Amanpreet Brar, Ana Minaya‐Bravo, Kerry A Breen, Chris Bretherton, Igor Lima Buarque, Joshua Burke, Edward J Caruana, Mohammad Chaar, Sohini Chakrabortee, Peter Christensen, Daniel Cox, Moises Cukier, Miguel F Cunha, Giana H Davidson, Anant Desai, Salomone Di Saverio, Thomas M Drake, John G Edwards, Muhammed Elhadi, Sameh Emile, Shebani Farik, Marco Fiore, J Edward Fitzgerald, Samuel Ford, Tatiana Garmanova, Gaetano Gallo, Dhruv Ghosh, Gustavo Mendonça Ataíde Gomes, Gustavo Grecinos, Ewen A Griffiths, Madalegna Gründl, Constantine Halkias, Ewen M Harrison, Intisar Hisham, Peter J Hutchinson, Shelley Hwang, Arda Isik, Michael D Jenkinson, Pascal Jonker, Haytham M.A. Kaafarani, Angelos Kolias, Schelto Kruijff, Ismail Lawani, Hans Lederhuber, Sezai Leventoglu, Andrey Litvin, Andrew Loehrer, Markus W Löffler, Maria Aguilera Lorena, Maria Marta Madolo, Piotr Major, Janet Martin, Hassan N Mashbari, Dennis Mazingi, Symeon Metallidis, Ana Minaya‐Bravo, Helen M Mohan, Rachel Moore, David Moszkowicz, Susan Moug, Joshua S Ng‐Kamstra, Mayaba Maimbo, Milagros Niquen, Faustin Ntirenganya, Maricarmen Olivos, Kacimi Oussama, Oumaima Outani, Marie Dione Parreno‐Sacdalanm, Francesco Pata, Carlos Jose Perez Rivera, Thomas D Pinkney, Willemijn van der Plas, Peter Pockney, Ahmad Qureshi, Dejan Radenkovic, Antonio Ramos‐De la Medina, Keith Roberts, April C Roslani, Martin Rutegård, Irène Santos, Sohei Satoi, Raza Sayyed, Andrew Schache, Andreas A Schnitzbauer, Justina O. Seyi‐Olajide, Neil Sharma, Richard Shaw, Sebastian Shu, Kjetil Soreide, Antonino Spinelli, Grant D Stewart, Malin Sund, Sudha Sundar, Stephen Tabiri, Philip Townend, Georgios Tsoulfas, Gabrielle H van Ramshorst, Raghavan Vidya, Dale Vimalachandran, Oliver J Warren, Duane Wedderburn, Naomi Wright, EuroSurg, European Society of Coloproctology (ESCP), Global Initiative for Children’s Surgery (GICS), GlobalSurg, GlobalPaedSurg, ItSURG, PTSurg, SpainSurg, Italian Society of Colorectal Surgery (SICCR), Association of Surgeons in Training (ASiT), Irish Surgical Research Collaborative (ISRC), Transatlantic Australasian Retroperitoneal Sarcoma Working Group (TARPSWG), Italian Society of Surgical Oncology (SICO).

#### 1.1.5 Collaborating authors

**Argentina**: Boccalatte LA (***Hospital Italiano de Buenos Aires***).

**Australia:** Batstone M, Hodge R (***Royal Brisbane and Women's Hospital***).

**Belgium**: Abeloos J, De Backer T, De Ceulaer J, Dick C, Diez‐Fraile A, Lamoral P, Spaas C (***AZ Sint‐Jan Brugge‐Oostende AV***); Schrijvers DLAL *(* ***Hospital Network Antwerp***); Willemse EBM (***Jules Bordet Institute***); Faris C, Maariën S, Van Haesendonck G, Van Laer C (***University Hospital Antwerp*** *);* Deron P (***University Hospital Gent***).

**Brazil**: Abdallah EA, Carvalho GB, Kowalski L, Vartanian J (***A.C. Camargo Cancer Center***); Gatti AP, Nardi CN, Oliva RNL (***Hospital Geral de Pirajussara***); Salem MC (***Irmandade da Santa Casa de Misericórdia de Porto Alegre***).

**Canada**: Cheng D, MacNeil D, Martin J, Mayer R, (***London Health Sciences Centre and St Josephs Health Care London***); Groot G (***Saskatoon City Hospital/Royal University Hospital/St. Paul's Hospital***).

**Colombia**: Acosta L, Mejia M, Perez CJ (***Fundacion Cardioinfantil‐IC***).

**Croatia**: Lorencin M, Luksic I, Mamic M (***University Hospital Dubrava***).

**Egypt**: Ashoush FM (***Alexandria Main University Hospital***); Osman NA (***Alexandria Medical Research Institute***); Safwat Shahine M (***Assiut University Hospital***); Eldaly A (***El‐Menshawy Hospital***); Elfiky MMA (***Kasr Alainy Hospital***); Amin A (***National Cancer Institute***); Elmorsi R, Refky B (***Oncology Center Mansoura University***); Essa MM (***Tanta University Hospital***).

**Ethiopia**: Mengistu G Mengesha (***Hawassa University Comprehensive Specialized Hospital***).

**France**: Dakpé s (***CHU Amiens***); Boucher S (***CHU Angers***); Ballouhey Q, Laloze J, Usseglio J (***CHU Limoges***); Hoffmann C (***Curie Institute***); Gregoire V (***Léon Bérard Cancer Center***); Lallemant B (***University Hospital of Nîmes***).

**Germany**: Blaurock M (***Greifswald University Medicine***); Reim D (***Klinikum Rechts der Isar TUM School of Medicine***); Boehm A (***Klinikum St. Georg, ENT Dept***.); Guntinas‐Lichius O (***Universitätsklinikum Jena***); Hölzle F, Modabber A, Winnand P (***University Hospital Aachen***); Kleeff J, Lorenz K, Ronellenfitsch U, Schneider R (***University Hospital Halle, Saale***); Betz CS, Böttcher A, Busch C, Möckelmann N (***University Medical Center Hamburg‐Eppendorf***); Inhestern JM (***Oberhavelkliniken Hennigsdorf***) Greve J, Hoffmann TK, Laban S, Vahl JM (***University Hospital Ulm***).

**Ghana**: Agyeman‐Prempeh A, Aning D, Barnor I, Darko‐Asante R, Dzogbefia M, Gaveh V, Gyimah D, Issahalq MD, Konney A, Poku M (***Komfo‐Anokye Teaching Hospital***); Adjeso T, Akornor ET, Amankwaa WO, Antwi DA, Apppiah‐Thompson P, Damah M, Kumi EO, Manan L, Murphy JP, Osei L, Setuagbe J (***Tamale Teaching Hospital***).

**Greece**: Arkadopoulos N, Danias N, Economopoulou P, Frountzas M, Kokoropoulos P, Larentzakis A, Michalopoulos NV, Nastos K, Parasyris S, Pikoulis E, Selmani J, Sidiropoulos TA, Vassiliu P (***Attikon University General Hospital***); Kalfountzos CE (***General Hospital of Larissa "Koutlimpaneio and Triantafylleio"***); Chatziioannou I, Corais C, Gkrinia E, Ntziovara A, Saratziotis A (***General University Hospital of Larissa***); Antoniadis K, Orestis O, Tatsis D (***George Papanikolaou General Hospital of Thessaloniki***); Baili E, Charalabopoulos A, Liakakos T, Schizas D, Spartalis E, Syllaios A, Zografos C (***Laiko University Hospital***).

**Guatemala**: Aguilera‐Arévalo M (***Hospital General San Juan De Dios***).

**India**: Misra S, Pareek P, Vishnoi J (***All India Institute of Medical Sciences, AIIMS, Jodhpur***); Chappity P, Kar M, Muduly DK, Sultania M (***All India Institute Of Medical Sciences, Bhubhaneshwar***); Agarwal S, Garg PK, Maharaj DD, Majumdar KS, Mishra N, Poonia D, Seenivasagam RK, Singh MP, Tiwari AR (***All India Institute Of Medical Sciences, Rishikesh***); Penumadu P (***Jawaharlal Institute of Postgraduate Medical Education and Research***); Rajan S (***King George's Medical University, Lucknow, UP***); Kumar S (***Max Superspeciality Hospital***); Raychowdhury R (***Ramakrishna Mission Seva Pratishthan***); Ghodke R (***Sanjay Clinic & Apollo Hospital***); Raychowdhury R (***Vivekananda Institute of Medical Sciences***).

**Ireland**: Barry C, Callanan D, Dias A, Haung L, Ionescu A, Sheahan P (***South Infirmary Victoria University Hospital***); Lennon P (***St. James's Hospital***).

**Israel:** Mizrachi A (***Rabin Medical Center***).

**Italy**: Deganello A (***ASST Spedali Civili, Ospedale di Brescia***); Pellini R, Pichi B (***Regina Elena National Cancer Institute***); Lemma F (***Azienda Ospedaliera di Padova***); Marino MV (***Azienda Ospedaliera, Ospedali Riuniti Villa Sofia, Cervello***); Bergonzani M, Varazzani A (***Azienda Ospedaliero‐Universitaria di Parma***); Bussu F, Perra T, Piras A, Porcu A, Rizzo D (***Cliniche San Pietro, A.O.U. Sassari***); Campisi G, Cordova A, Franza M, Rinaldi G, Toia F (***Department of Surgical, Oncological and Oral Sciences, University of Palermo***); Gianni A (***Fondazione IRCCS Ca' Granda ‐ ospedale Maggiore Policlinico***); Giannini L (***Fondazione IRCCS Istituto Nazionale dei Tumori, Milano***); Gordini L (***Fondazione Policlinico Universitario Agostino Gemelli***); Baldini E, Conti L (***G. Da Saliceto***); De Virgilio A, Ferreli F, Gaino F, Mercante G, Spriano G (***Humanitas University; IRCCS Humanitas Research Hospital***); Ansarin M, Chu F, De Berardinis R, Ietrobon G, Tagliabue M (***Istituto Europeo di Oncologia ‐ IRCCS ‐Milano***); Ionna F (***Istituto Nazionale Tumori Fondazione, Pascale ‐ IRCCS***); Baietti A, Maremonti P, Neri F, Prucher G, Ricci S (***Ospedale Maggiore/Bellaria Carlo Alberto Pizzardi AUSL Bologna***); Casaril A, Nama M (***Ospedale Pederzoli***); Cotoia A, Lizzi V, Vovola F (***Ospedali Riuniti Azienda Ospedaliera Universitaria Foggia***); Bruzzaniti P, Familiari P, Lapolla P, Marruzzo G, Mingoli A, Ribuffo D (***Policlinico Umberto I***); Cipriani R, Contedini F, Lauretta M, Marchetti C, Melotti M, Pignatti M, Pinto V, Pizzigallo A, Ricotta F, Tarsitano A (***Sant'Orsola Hospital, Alma Mater Studiorum University of Bologna, Italy***); Catarzi L, Consorti G (***University Hospital Umberto, Ancona***).

***Libya***: Abdulwahed EA, Alshareea EA, Biala MI, Ghmagh RJ (***Tripoli Central Hospital***).

**Malaysia**: Ibrahim AF (***Sarawak General Hospital***); Liew YT (***University Malaya Medical Centre***).

**Mexico**: Alvarez MR, Arrangoiz R, Cordera F, Gómez‐Pedraza A (**ABC Medical Center**); Soulé‐Martínez CE (***Hospital Central Norte Pemex***); Becerril OS (***Hospital Juárez de México***); Becerra GFC (***Hospital San Angel Inn Patriotismo***).

**Morocco**: Arkha Y, Bechri H, El Ouahabi A, Oudrhiri M (***Hôpital des Spécialités ‐ ONO***); Benkabbou A, Majbar M, Mohsine R, Souadka A (***Universite Mohammed V Institut National d'Oncologie Rabat***).

**Nepal**: Lageju N (***Nepal Police Hospital***).

**Netherlands**: Schreuder WH (***Antoni van Leeuwenhoek Ziekenhuis***); Hardillo J (***Erasmus Medisch Centrum***); de Bree R (***University Medical Center Utrecht***); Schweitzer D (***Zuyderland Medical Center***).

**Nigeria**: Adeyeye AA, Enoch EE (***Afe Babalola University Multi‐System Hospital***); Sholadoye TSTT (***Ahmadu Bello University Teaching Hospital***); Wuraola F (***Obafemi Awolowo University Teaching Hospitals Complex***); Oyelakin O (***University College Hospital, Ibadan***).

**Pakistan**: Khokhar MI (***Lahore General Hospital, PGMI, AMC***); Ayub B (***Patel Hospital***).

**Poland**: Walędziak M (**Military Institute Of Medicine**); Szewczyk M (***The Gerater Poland Cancer Center***).

**Portugal**: Faria C (***Centro Hospitalar Sao Joao***); Cardoso P (***Hospital Faro, Centro Hospitalar Universitario do Algarve***); Castro Silva J (***IPO Porto***).

**Saudi Arabia**: AlKharashi E (***Prince sultan military medical city***).

**Serbia**: Jelovac D, Petrovic M, Sumrak S (***Clinic for Maxillofacial Surgery, School of Dental Medicine, University of Belgrade***); Asceric RR, Bojicic JM, Kovacevic BM, Krdzic ID, Milentijevic MA, Milutinovic VZ, Stefanovic ZB (***Zvezdara University Medical Center***).

**Spain**: Villacampa JM (***Fundación Jimenez Diaz University Hospital***); Jiménez Carneros V, Salazar Carrasco A, Carabias Hernandez A, Alonso Lamberti L, León Ledesma R, Jiménez Miramón FJ, Jover Navalón JM, Garcia Quijada J, Ramos Rodriguez J, Valle Rubio A (***Getafe University Hospital***); Vilaseca I (***Hospital Clínic of Barcelona***); Escartin J (***Hospital Royo Villanova***); Estaire‐Gomez M, Padilla Valverde D (***Hospital General Universitario de Ciudad Real***); Tousidonis M (***Hospital General Universitario Gregorio Marañón***); Lopez F (***Hospital Universitario Central de Asturias, HUCA***); Deandrés‐Olabarria U, Durán‐Ballesteros M, Fernández‐Pablos F, Ibáñez‐Aguirre F, Sanz‐Larrainzar A, Ugarte‐Sierra B (***Hospital Universitario de Galdakao***); Di Martino M, Prada J (***Hospital Universitario de la Princesa***); Jariod‐Ferrer UM (***Hospital Universitario Miguel Servet***); Landaluce Olavarria A (***Hospital Urduliz***); Rey‐Biel J (***Rey Juan Carlos University Hospital***); Díaz de Cerio P (**Saint Peter Hospital**); Sánchez Barrueco A (***Villalba University General Hospital***).

**Sweden**: Lindqvist EK (***Karolinska University Hospital***); Sund M (***Umea University Hospital***).

**Switzerland**: Piantanida R (***Ente Ospedaliero Cantonale***); Giger R, Hool S, Müller SA (***Inselspital, Bern University Hospital, University of Bern***); Stoeckli SJ (***Kantonsspital St. Gallen***); Simon C (***Lausanne University Hospital CHUV***).

**Syrian Arab Republic**: Toutounji T (***Aleppo University Hospital***);Al assaf A (***Al‐Mouwasat University Hospital***); Hammed AM, Hammed SM, Mahfoud M (***Tishreen University Hospital***).

**Turkey**: Arikan A (***Acibadem Maslak Hospital***); Yalkin Ö (***Ali Osman Sönmez Oncology Hospital***); İflazoğlu N (***Bursa City Hospital***); Isik A, Leventoglu S (***Erzincan University Hospital***); Aydemir L, Basaran B, Sen C, Comert ‐Ulusan M (***Istanbul University Faculty of Medicine***); Basaran B (***Karaman Training and Research Hospital***); Saracoglu KT (***Kartal Dr. Lutfi Kirdar Training and Research Hospital***); Saracoglu A (***Marmara University, School of Medicine***); Mantoglu B (***Sakarya Faculty Of Medicine***); Kucuk G (***Samsun Training and Research Hospital***); Aygun N, Baran E, Tanal M, Eray Tufan A, Uludag M, Gürkan Yetkin S, Yigit B (***Sisli Hamidiye Etfal Training and Research Hospital***); Calik B, Demirli Atici S, Kaya T (***University of Health Sciences Tepecik Training and Research Hospital***).

**Uganda**: Sikakulya FK (***Kampala International University Teaching Hospital***).

**United Arab Emirates**: Abdel‐Galil KMAH (***Tawam Johns Hopkins Hospital***).

**United Kingdom**: Lowe T (***Aberdeen Royal Infirmary***); Durrani AJ, Habeeb A, Irune E, Luke L, Masterson L, Murphy SH, Segaren N, Walker C, Waseem S (***Addenbrooke's Hospital***); Jones TM, Loh C, Pringle S, Schache AG, Shaw RJ (***Aintree University Hospital***); Stenhouse J (***Altnagelvin Area Hospital***); Armstrong M (***Borders General Hospital***); Sood S, Sutton D (***Bradford Royal Infirmary***); Thomas S (***Bristol Royal Infirmary***); Clarke P (***Charing Cross Hospital***); Winter SC (**Churchill Hospital**); Hislop S (***Crosshouse University Hospital***); Counter PR (***Cumberland Infirmary***); Ghazali N (***East Lancashire Hospitals NHS Trust***); Lloyd C (***Glan Clwyd Hospital***); Prabhu V (***Glangwili General Hospital***); Godden D, Whitley S (***Gloucestershire Royal Hospital***); Butler C, Nash R (***Great Ormond Street Hospital for Children***); El‐Boghdadly K, Fry A, Niziol R (***Guy's Hospital***); De M, Gill CK (***Heartlands Hospital***); Crank S (***Hull University Teaching Hospitals NHS Trust***); Mace AD (***Imperial College NHS Trust***); Ho M (***Leeds General Infirmary***); Mair M (***Leicester Royal Infirmary***); Kothari P (***Luton and Dunstable University Hospital***); Homer J, Sainuddin S (***Manchester Royal Infirmary***); Egan RJ, Kittur M (***Morriston Hospital Swansea***); Burgess C (***Musgrove Park Hospital)***; O'Hara J (***Newcastle Upon Tyne Hospitals NHS Foundation Trust***); Manickavasagam J, McDonald C (**Ninewells Hospital**); Burrows S (***Norfolk and Norwich University Hospital***); Java KR, Katre C (***North Manchester General Hospital***); Ahmed A, Siddique H (***Northwick Park Hospital***); King E, Ramchandani P (***Poole Hospital***); Naredla PR (***Princess Royal Hospital***) Brennan P, Ringrose T, Schmidt F (***Queen Alexandra Hospital***); Mak JKC, Nankivell P, Parmar S, Sharma N (***Queen Elizabeth Hospital Birmingham***); Douglas C, McCaul J, McCaul J (***Queen Elizabeth University Hospital***); Dhanda J (***Queen Victoria Hospital***); Ghazali N, Kyzas P, Vassiliou L (***Royal Blackburn Hospital***); Kumar A (***Royal Derby Hospital***); Husband A (***Royal Devon and Exeter Hospital***); Hulbert J (***Royal Glamorgan Hospital***); Ingrams D, Parkin R (***Royal Gwent Hospital***); Varley I (***Royal Hallamshire Hospital***); Gahir D, George A, Zakai D (***Royal Stoke University Hospital***); Bater M (***Royal Surrey County Hospital***); Surwald C (***Royal Sussex County Hospital***); Devlin B, Leonard CG (***Royal Victoria Hospital***); Pigadas N, Snee D (***Royal Wolverhampton NHS Trust***); Singh RP (***Southampton General Hospital***); Hyde NC (***St George's Hospital***); Paley M (***St John's hospital, Livingston***); Cocks H, Wilson A (***Sunderland Royal Hospital***); Choi D (***The National Hospital for Neurology and Neurosurgery***); Kerawala CJ, Riva F (***The Royal Marsden NHS Foundation Trust***); Dickason A (***Torbay and South Devon NHS Trust***); Semple CJ (***Ulster Hospital Dundonald***); Schilling C (***University College London Hospital at Westmoreland Street***); Naredla PR, Walton G (***University Hospitals Coventry and Warwickshire NHS Trusts***); Rees‐Stoner O (***University Hospitals Plymouth***); Scott N (***University Hospital of Wales***); Nixon IJ (***Western General Hospital***); Tighe D (***William Harvey Hospital***); Mattine S (***Worcestershire Royal Hospital***); Chu MMH, Pothula V (***Wrightington, Wigan & Leigh NHS Foundation Trust***).

**United States**: Lee W (***Duke University Medical Center***); Brown L, Ganly I (***Memorial Sloan Kettering Cancer Center***); Alpert N, Illezeau CN , Miles B, Rapp J, Taioli E (***Mount Sinai Hospital***); Azam MT, Choudhry AJ, Marx W (***SUNY Upstate University Hospital***); Stein J (***The University Hospital***); Ying Y (***University of Alabama Birmingham***); Gross ND (***University of Texas MD Anderson Cancer Center***); Almasri M, Joshi R, Kulkarni G, Marwan H, Mehdi M (***University of Texas Medical Branch***); Sumer B (***UT Southwestern Medical Center***).
