# Supplementary material for: UK Head and neck cancer surgical capacity during the second wave of the COVID—19 pandemic: Have we learned the lessons? COVIDSurg collaborative
Source: Clin Otolaryngol. 2021 Mar 29;46(4):729–35. doi: 10.1111/coa.13749 (PMC8014442; doi:10.1111/coa.13749)
Supplement: Supplementary file 1 — Supplementary Material [file COA-46-729-s002.docx]

**Appendix: Authorship COVIDSurg Collaborative***

*** Collaborators (PubMed-citable):**

*Writing group*

Richard Shaw, Andrew G Schache, Michael Wing Sung Ho, Stuart C Winter, James Glasbey, Ian Ganly, Martin Batstone, Juan Rey Biel, Paul C Nankivell, Christian Simon, Omar Omar, Joana FF Simoes, Dmitri Nepogodiev, Aneel Bhangu, Tom Pinkney, Laura McGill, Rita Perry, Terry Hughes

*CovidSurg Operations Committee*

Kwabena Siaw-Acheampong, Ruth A Benson, Edward Bywater, Daoud Chaudhry, Brett E Dawson, Jonathan P Evans, James C Glasbey, Rohan R Gujjuri, Emily Heritage, Conor S Jones, Sivesh K Kamarajah, Chetan Khatri, Rachel A Khaw, James M Keatley, Andrew Knight, Samuel Lawday, Elizabeth Li, Harvinder S Mann, Ella J Marson, Kenneth A McLean, Siobhan C Mckay, Emily C Mills, Dmitri Nepogodiev, Gianluca Pellino, Maria Picciochi, Elliott H Taylor, Abhinav Tiwari, Joana FF Simoes, Isobel M Trout, Mary L Venn, Richard JW Wilkin, Aneel Bhangu.

*International Cancer Leads (*denotes specialty Principal Investigator)*

James C Glasbey (*Chair*); Head and neck: Richard Shaw*, Andrew G Schache, Stuart C Winter, Michael W S Ho, Paul Nankivell, Juan Rey Biel, Martin Batstone, Ian Ganly, Christian Simon.

*Dissemination Committee*

Joana FF Simoes (*Chair*); Tom EF Abbott, Michel Adamina, Adesoji O Ademuyiwa, Arnav Agarwal, Ehab Alameer, Derek Alderson, Felix Alakaloko, Markus Albertsmeiers, Osaid Alser, Muhammad Alshaar, Sattar Alshryda, Alexis P Arnaud, Knut Magne Augestad, Faris Ayasra, José Azevedo, Brittany K Bankhead-Kendall, Emma Barlow, Ruth A Benson, Ruth Blanco-Colino, Amanpreet Brar, Ana Minaya-Bravo, Kerry A Breen, Chris Bretherton, Igor Lima Buarque, Joshua Burke, Edward J Caruana, Mohammad Chaar, Sohini Chakrabortee, Peter Christensen, Daniel Cox, Moises Cukier, Miguel F Cunha, Giana H Davidson, Anant Desai, Salomone Di Saverio, Thomas M Drake, John G Edwards, Muhammed Elhadi, Sameh Emile, Shebani Farik, Marco Fiore, J Edward Fitzgerald, Samuel Ford, Tatiana Garmanova, Gaetano Gallo, Dhruv Ghosh, Gustavo Mendonça Ataíde Gomes, Gustavo Grecinos, Ewen A Griffiths, Madalegna Gründl, Constantine Halkias, Ewen M Harrison, Intisar Hisham, Peter J Hutchinson, Shelley Hwang, Arda Isik, Michael D Jenkinson, Pascal Jonker, Haytham MA Kaafarani, Angelos Kolias, Schelto Kruijff, Ismail Lawani, Hans Lederhuber, Sezai Leventoglu, Andrey Litvin, Andrew Loehrer, Markus W Löffler, Maria Aguilera Lorena, Maria Marta Madolo, Piotr Major, Janet Martin, Hassan N Mashbari, Dennis Mazingi, Symeon Metallidis, Ana Minaya-Bravo, Helen M Mohan, Rachel Moore, David Moszkowicz, Susan Moug, Joshua S Ng-Kamstra, Mayaba Maimbo, Milagros Niquen, Faustin Ntirenganya, Maricarmen Olivos, Kacimi Oussama, Oumaima Outani, Marie Dione Parreno-Sacdalanm, Francesco Pata, Carlos Jose Perez Rivera, Thomas D Pinkney, Willemijn van der Plas, Peter Pockney, Ahmad Qureshi, Dejan Radenkovic, Antonio Ramos-De la Medina, Keith Roberts, April C Roslani, Martin Rutegård, Irène Santos, Sohei Satoi, Raza Sayyed, Andrew Schache, Andreas A Schnitzbauer, Justina O. Seyi-Olajide, Neil Sharma, Richard Shaw, Sebastian Shu, Kjetil Soreide, Antonino Spinelli, Grant D Stewart, Malin Sund, Sudha Sundar, Stephen Tabiri, Philip Townend, Georgios Tsoulfas, Gabrielle H van Ramshorst, Raghavan Vidya, Dale Vimalachandran, Oliver J Warren, Duane Wedderburn, Naomi Wright, EuroSurg, European Society of Coloproctology (ESCP), Global Initiative for Children’s Surgery (GICS), GlobalSurg, GlobalPaedSurg, ItSURG, PTSurg, SpainSurg, Italian Society of Colorectal Surgery (SICCR), Association of Surgeons in Training (ASiT), Irish Surgical Research Collaborative (ISRC), Transatlantic Australasian Retroperitoneal Sarcoma Working Group (TARPSWG), Italian Society of Surgical Oncology (SICO).

*Collaboratoring authors (*denotes Site Principal Investigators)*

TBC 8-2-21
