# Supplementary material for: UK Head and neck cancer surgical capacity during the second wave of the COVID—19 pandemic: Have we learned the lessons? COVIDSurg collaborative
Source: Clin Otolaryngol. 2021 Mar 29;46(4):729–35. doi: 10.1111/coa.13749 (PMC8014442; doi:10.1111/coa.13749)
Supplement: Supplementary file 2 — Supplementary Material [file COA-46-729-s001.pdf]

Resize font:

Head & Neck Cancer Global Service Impact Survey (COVIDSurg-Cancer Sites)

The consequences of the COVID pandemic on patients are becoming clear through the collaborative efforts of the COVIDSurg group and we are grateful for your efforts in recording data on patients in your hospital.

Many parts of the world are now experiencing a further & significant COVID-19 case surge – we want to find out what has changed since March 2020 – so here is a very short survey about the capacity and safety of head and neck cancer surgery. This REDCAP survey will close Sunday 7th Feb – we have a plan for a **very fast** turnaround of this snapshot survey.

We would ask you to complete this BRIEF questionnaire assessing the impact of COVID on the service and care pathways.

The questionnaire will allow us to assess both national and global variations in H&N care access.

One reply is required per centre with no patient data. Please do respond – it will take 2 minutes and we will publish the findings recognising all contributors in authorship (as has been the case for COVIDSurg publications).

If you are not registered as a COVIDSurg site then please STILL ENTER A RESPONSE for your hospital. You can enter your hospitals details in the dropdown fields.

TO ALLOW RAPID PUBLICATION OF FINDINGS - PLEASE COMPLETE THE QUESTIONNAIRE BEFORE Sunday 7th February 2021.

Finally, initial details for the COVIDSurg Cancer H&N Follow-on study will be released soon. We'll be asking for 1-year and 2-year data on the patients that each COVIDSurg site included during the period March-June 2020. The first data collection timepoint will be in June 2021 (1 year data).

You can find out what we do with your data in our Privacy Notice.

Hospital details

Hospital Name:

Please start typing hospital name in the box to search the list of hospitals.

If your hospital is not listed please select "Other".

* must provide value

If there is an error in the name of your hospital let us know by emailing BhamRed@contacts.bham.ac.uk

Country

* must provide value

Please only complete ONE survey per site/hospital
Ideally completed by the Lead Head and neck Surgeon or CovidSurg-Cancer PI.

Person Completing Survey

First Name	Last Name	Email address

Please complete each of the elements below giving an estimate for YOUR SERVICE/HOSPITAL AS A WHOLE

For each question, the estimates are for three distinct timepoints;

- **Pre COVID-19 (eg. Early 2019)**
- **March-June 2020 (when the 1st peak of infections was prevalent worldwide)**
- **Last 7 Days**

Service Pressure, Capacity

	Pre COVID-19	March - June 2020	Last 7 Days
	Percentage(%)		
**Head & Neck Cancer Referrals (%)**<br>Estimate the % of normal Pre-pandemic H&N cancer referrals received per week	**100**		
**Theatre Capacity (%)**<br>Estimate the percentage of normal Theatre Capacity (% of full H&N theatre capacity)	**100**		
**Outpatient Face to Face appointments (%)**<br>Estimate the % of clinic appointments that are undertaken face to face			

**Elective critical care bed availability for post operative H&N cases**<br>Estimate the capacity of Elective critical care bed availability for post operative H&N cases	
**Pre COVID-19**	
**March - June 2020**	
**Last 7 Days**	

		March - June 2020	January - February 2021
**Have you moved the site of Surgery for Head and Neck Cancer for any patients in the time period**	**Different site, same surgical team**		
	**Different site, different surgical team**		

Restriction in service capacity

	Pre COVID-19	March - June 2020	Last 7 Days
	Percentage (%)		
**Delay**<br>Estimate the % of cases delayed due to restriction in service capacity			
**Therapeutic migration to non-surgical care**<br>Estimate the % of cases migrated due to restriction in surgical service capacity (e.g. A patient receiving radiotherapy instead of surgery for SCC larynx)	**0**		
**De-escalation of surgery**<br>Estimate the % of cases where the surgical treatment delivered was compromised from normal standard of care	**0**		

COVID Testing, Infection rates and Vaccination

	Pre COVID-19	March - June 2020	Last 7 Days
	Percentage (%)		
**Preoperative COVID Testing**<br>Estimate the percentage of H&N cancer cases for which COVID Testing was undertaken (< 72 hours before surgery)	0		
**Cumulative proportion of H&N Surgical team members tested positive for COVID-19**<br>Estimate the % of H&N Surgical team members who have been COVID+ (tested or presumed positive)	0		
**Full PPE**<br>Estimate the % of procedures undertaken using Full PPE (FFP3 or equivalent masks usage for H&N operating)	0		
**Vaccination of H&N Surgical team members**<br>Estimate the % of H&N Surgical team members who have been vaccinated with at least 1 dose against SARS-CoV-2	0	0	
**Routine (at least weekly) staff testing**<br>Estimate the % of H&N team members who are rountinely (at least weekly) tested against SARS-CoV-2, any type of tests used	0		

Please make any other relevant comments about your current H&N Surgery Service

Expand

Submit
